# Epidemiological characteristics of paediatric COVID-19 and influenza co-infections in the United States, 2020–2024

**DOI:** 10.1017/S0950268826101915

**Published:** 2026-07-29

**Authors:** Ozair H. Naqvi, Aaron M. Wendelboe, William H. Beasley, Donna L. Tyungu, Kai Ding, Katrin G. Kuhn, James J. Cheng, A. Jared Anzalone, Amanda E. Janitz

**Affiliations:** 1 Department of Biostatistics and Epidemiology, Hudson College of Public Health, University of Oklahoma Health Sciences, Oklahoma City, Oklahoma, USA; 2 Department of Epidemiology, Fay W. Boozman College of Public Health, University of Arkansas for Medical Sciences, Little Rock, Arkansas, USA; 3 Department of Pediatrics, University of Oklahoma College of Medicine, University of Oklahoma Health Sciences, Oklahoma City, Oklahoma, USA; 4 Department of Biostatistics, College of Public Health, University of Nebraska Medical Center, Omaha, Nebraska, USA

**Keywords:** co-infection, COVID-19, influenza, paediatrics

## Abstract

Evidence describing paediatric COVID-19 and influenza co-infection is limited because influenza circulation was minimal during the early COVID-19 pandemic. We used the National Clinical Cohort Collaborative, a multicentre electronic health record repository, to describe the characteristics and clinical outcomes of paediatric COVID-19 and influenza co-infections in the United States from March 2020–April 2024. We defined co-infection as COVID-19 and influenza identified <7 days apart. We reported demographics, pre-existing diagnoses, and clinical outcomes and used generalized estimating equation models. We defined clinical outcomes as Severe/Moderate disease (hospitalization, critical care, or death) versus emergency department/outpatient encounters. We repeated analyses using varying co-infection definitions to assess robustness of findings. Among 1.53 million patients, 10,277 were co-infected, and 647 (6%) were hospitalized. Co-infections occurred predominantly among children aged <12 years. The most common pre-existing diagnoses were obesity (24%), asthma (10%), and congenital/genetic conditions (5%). The risk for Severe/Moderate disease was greatest among persons aged 0–4 years (adjusted risk ratio (aRR) 3.05; 95% confidence interval (CI): 2.35–3.97) and those with ≥2 pre-existing diagnoses (aRR 2.89; 95% CI: 2.43–3.43). Similar distributions of disease severity were observed across varying co-infection definitions. This study provides a large-scale epidemiological characterization of paediatric COVID-19 and influenza co-infection and successfully implemented a practical EHR-based co-infection case definition for future analyses.

## Background

Co-infections of respiratory diseases among paediatric patients are known to prolong illness and lead to more frequent health care use [1]. During the first 24 months of the COVID-19 pandemic, influenza and other infections such as respiratory syncytial virus (RSV) were suppressed, likely a result of non-pharmaceutical interventions (NPIs) to prevent COVID-19 transmission [2]. In 2022, influenza activity in the United States (US) returned to pre-pandemic levels and was characterized as ‘high severity’ among children and adolescents for only the third time since 2009 [3]. Influenza contributes to paediatric morbidity and mortality in the United States, with an average annual estimate of 113 influenza-associated paediatric deaths [4], while COVID-19 has been attributed to 1,879 provisional paediatric deaths as of May 31, 2024 [5]. During the 2022–2023 influenza season, the Centers for Disease Control and Prevention (CDC) estimated that 6% of paediatric influenza-associated hospitalizations were reportedly co-infected with SARS-CoV-2 [6]. A higher proportion of these co-infected patients received invasive mechanical ventilation (IMV) compared to those with influenza only (13% vs. 4%) [6]. Co-infected cases also made up 16% of influenza-associated paediatric deaths [6].

Many published studies exploring COVID-19 and influenza co-infection (hereby referred to as ‘co-infection’) among paediatric populations were conducted during the 2020–2021 and 2021–2022 influenza seasons, when influenza activity was minimal [2]. Consequently, the number of reported paediatric co-infections was low [7, 8]. Studies during these seasons provide limited evidence in making inferences related to clinical and public health practice, since influenza has returned to pre-pandemic levels. Evidence has indicated that more data are needed to assess risk factors and clinical outcomes associated with co-infections [9]. Ambiguous and inconsistent definitions of co-infection also limit a clear understanding of the clinical and public health impact of co-infection [10]. This includes variation in how studies distinguish co-infection from other types of infection, as well as differences in how respiratory pathogens are identified [10].

We used the National Clinical Cohort Collaborative (N3C), one of the largest integrated sources of electronic health record (EHR) data on COVID-19 clinical outcomes in the US [11] to investigate the epidemiology of COVID-19 and influenza co-infection among children, adolescents, and young adults from March 2020 to April 2024. Specifically, we aimed to quantify the proportion of patients aged ≤21 years with COVID-19 and influenza co-infection; describe the demographic characteristics, pre-existing medical conditions, and clinical outcomes of co-infected patients; and assess changes in co-infection detections and disease severity following the re-emergence of influenza circulation in 2022. In addition, we examined associations between age group and medical complexity with clinical outcomes among co-infected patients and evaluated the robustness of a reproducible EHR-based working case definition for co-infection using alternative specification thresholds.

## Methods

### Data source and study design

A retrospective observational study of patients aged ≤21 years in the N3C (*n* = 11,562) was performed to assess the overall burden and clinical outcomes associated with COVID-19 and influenza co-infection from March 2020 to April 2024. We followed the Enhancing the Quality and Transparency of Health Research (EQUATOR) reporting and Reporting of Studies Conducted Using Observational Routinely Collected Health Data (RECORD) guidelines [12].

The N3C Data Enclave collects and harmonizes longitudinal EHR data across over 80 data partners in the United States (Supplementary eMethods) [13]. The N3C includes data on persons with a positive SARS-CoV-2 laboratory test (polymerase chain reaction (PCR), antigen (Ag)) or a COVID-19 diagnostic code as well as matched non-cases (2:1 non-COVID-19: COVID-19) based on data partner, age, sex, race, and ethnicity.

We used N3C to collect data on patients with COVID-19 based on two criteria: (1) SARS-CoV-2 testing (LOINC codes) and (2) diagnostic condition codes (ICD-10-CM and SNOMED CT). We identified patients in the N3C with influenza in the same way, using LOINC, ICD-10-CM and SNOMED CT codes [14]. We did not restrict inclusion based on symptom status; therefore, asymptomatic or screening-identified cases may have been included. We used the N3C definition of index dates, defined as a patient’s first encounter involving COVID-19 or influenza (laboratory result or diagnostic code) [15]. For patients with multiple qualifying encounters or test results, the first documented COVID-19 index date and first documented influenza index date were used; subsequent encounters were used only to classify associated clinical outcomes within the defined outcome window. We defined co-infection as a patient with an index date for COVID-19 and influenza seven or fewer days apart. This definition was based on similar studies of paediatric co-infections involving COVID-19 [16, 17]. We excluded data partners that did not report minimal data elements (*n* = 12; Supplementary eTable1) and conducted a complete case analysis; therefore, individuals with unknown values for sex, race, and ethnicity were excluded (*n* = 1,285; Supplementary eTable2). We assessed our choice of complete case analysis by generating selection probabilities and comparing crude outcomes between those included and excluded (Supplementary eTable3) [18].

### Patient measurements

We incorporated patient age group (at infection) based on CDC’s criteria used to assess infection-associated hospitalizations among children, adolescents, and young adults (0–4 years, 5–11 years, 12–17 years, or 18–21 years) [19]. We also categorized sex (male or female) and race/ethnicity (American Indian/Alaska Native, Asian, Black/African American, Hispanic or Latino, Native Hawaiian or Other Pacific Islander, Other, and White). We defined and assigned pre-existing diagnoses to patients using the Paediatric Complex Chronic Conditions (PCCC) [20] and certain comorbidities in the N3C (asthma [21]; obesity [22, 23]; and diabetes mellitus, types 1 or 2) (Supplementary eTable4) [24, 25]. If a patient had no documentation of a pre-existing diagnosis, the corresponding variable would be indicated as ‘no’. To assess the impact of medical complexity on severe outcomes, we calculated the frequency and percentage of patients with one, two or more, or no pre-existing diagnoses documented. See Supplementary eTable4 for details on all patient measurement concept set definitions.

We defined two pandemic phases, ‘Phase 1’ and ‘Phase 2’, to denote the periods before and after the re-emergence of influenza in 2022 [26]. The first phase was defined as 1 March 2020 to 31 March 2022, which included the initial emergence of the Omicron strain; the second phase included the period from 1 April 2022 to 30 April 2024, which has largely been dominated by subvariants of the Omicron strain [27]. We were secondarily interested in assessing co-infection detections by influenza season and SARS-CoV-2 variant period of predominance.

### Clinical outcomes

We defined COVID-19 and influenza-associated visits as patient encounters in the N3C with either disease documented in the EHR from 14 days before to 14 days after the index date, aligning with CDC guidelines [28, 29]. Hospitalized patients were flagged for severe disease if they had any of the following documented during their visit: use of vasopressors/inotropes, IMV, extracorporeal membrane oxygenation (ECMO), or death ((inpatient death or discharge to hospice) recorded within 60 days following the index date). Patients with no recorded emergency department (ED) or hospital visit were categorized as outpatients.

We assigned disease severity progression as Mild (outpatient only), Mild ED (outpatient with ED visit), Moderate (hospitalized without requiring IMV, vasopressors/inotropes, or ECMO), and Severe (hospitalized and requiring IMV, vasopressors/inotropes, ECMO, or death) using an adapted version of the Clinical Progression Scale (CPS) established by the World Health Organization (WHO) and used in prior N3C paediatric studies [30].

### Statistical analysis

We restricted patients to those with co-infections to investigate the change in demographics, pre-existing diagnoses, and clinical outcomes by pandemic phase. We calculated descriptive statistics using frequencies and proportions for patients by age group, sex, race and ethnicity, pre-existing diagnoses, and clinical outcomes. We then investigated the relationship between age group, number of pre-existing diagnoses, and clinical outcomes. We conducted statistical testing using generalized estimating equations (GEE) with a binomial distribution and log link function to calculate the risk ratios (RR) and 95% confidence intervals (CI) of each variable of interest using the geepack package in R [31]. All models used data partner as a clustering variable to account for differences in N3C site reporting. We modelled Severe/Moderate disease versus Mild ED/Mild outpatient only (reference group). We also assessed statistical interaction between each exposure variable and pandemic phase. If an interaction was detected (*p* < 0.05), we would stratify models by pandemic phase. To evaluate confounding, we used a change-in-estimate approach implemented through backward elimination, retaining variables that changed the primary exposure parameter estimate by >10%. Finally, we conducted two sensitivity analyses: (1) to compare the sensitivity, specificity, and positive predictive value (PPV) of our co-infection case definition compared to a gold standard (positive COVID-19 and influenza laboratory results documented on the same day) and (2) to explore the impact of varying co-infection case definitions on our main study results. We used an alpha level of 0.05 for all statistical testing.

All analyses were performed within the N3C Data Enclave using R v4.1 in accordance with N3C privacy and download review policies [11]. This retrospective observational study received Institutional Review Board (IRB) approval from the University of Oklahoma Health Sciences Center (OUHSC) (#16868) was conducted under reliance on a centralized IRB at Johns Hopkins University (#00249128). The OUHSC IRB determined this study did not meet the criteria for human subjects research. The N3C Data Access Committee reviewed and approved the limited data set used for this study (DUR RP-0585B0).

## Results

### Patient demographics

**Figure 1.**
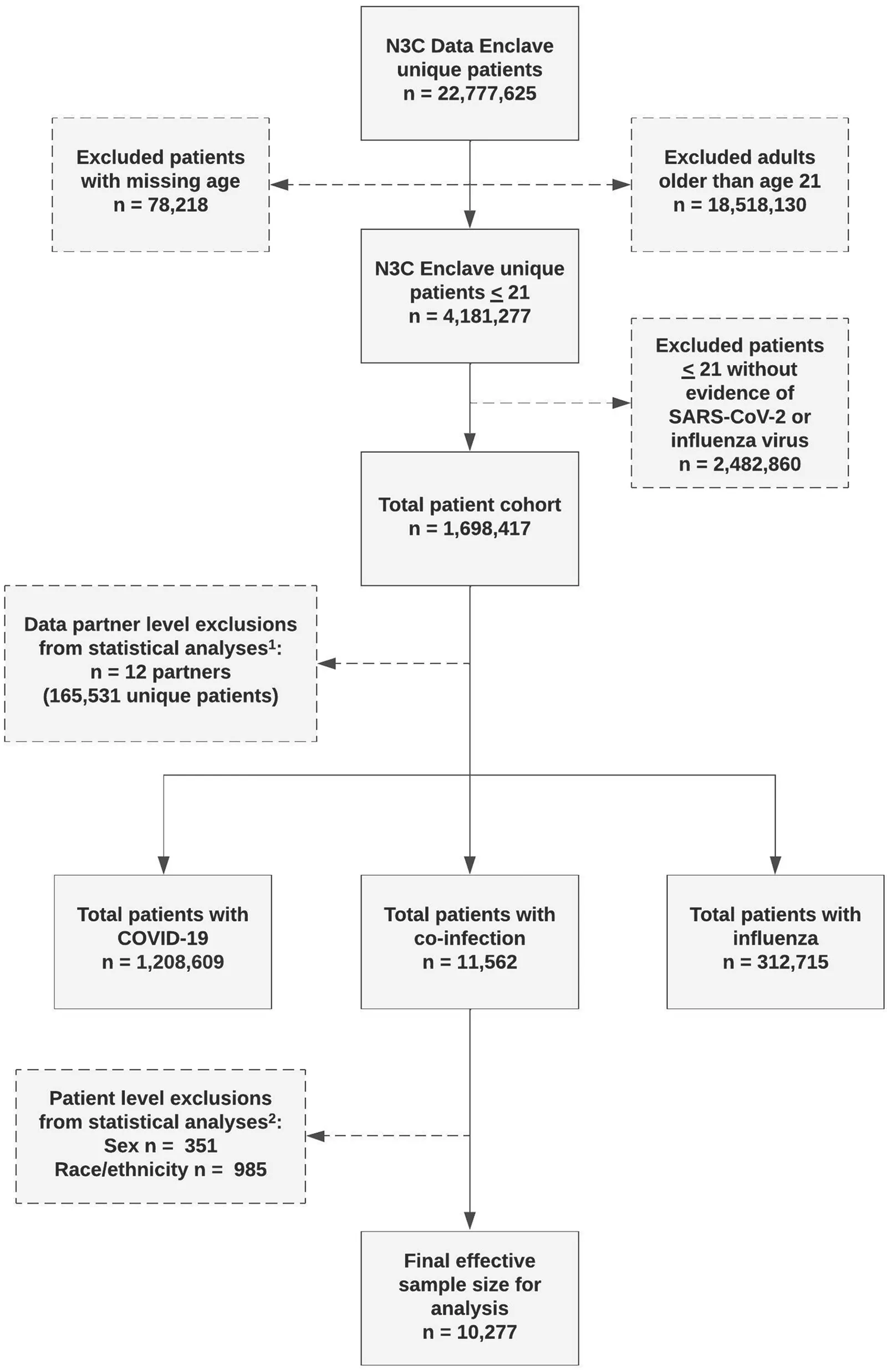
Flow diagram for study cohort, National Clinical Cohort Collaborative (N3C), March 2020–April 2024. If a data partner did not report any of the key data elements, they were excluded from the study. Quality among each N3C data partner based on the Observational Health Data Science and Informatics (OHDSI) framework in Dixon et al. (2020) [32] Missing variable counts are not mutually exclusive, total unique patients excluded *n* = 1,285. Figure 1. long description.

**Figure 2.**
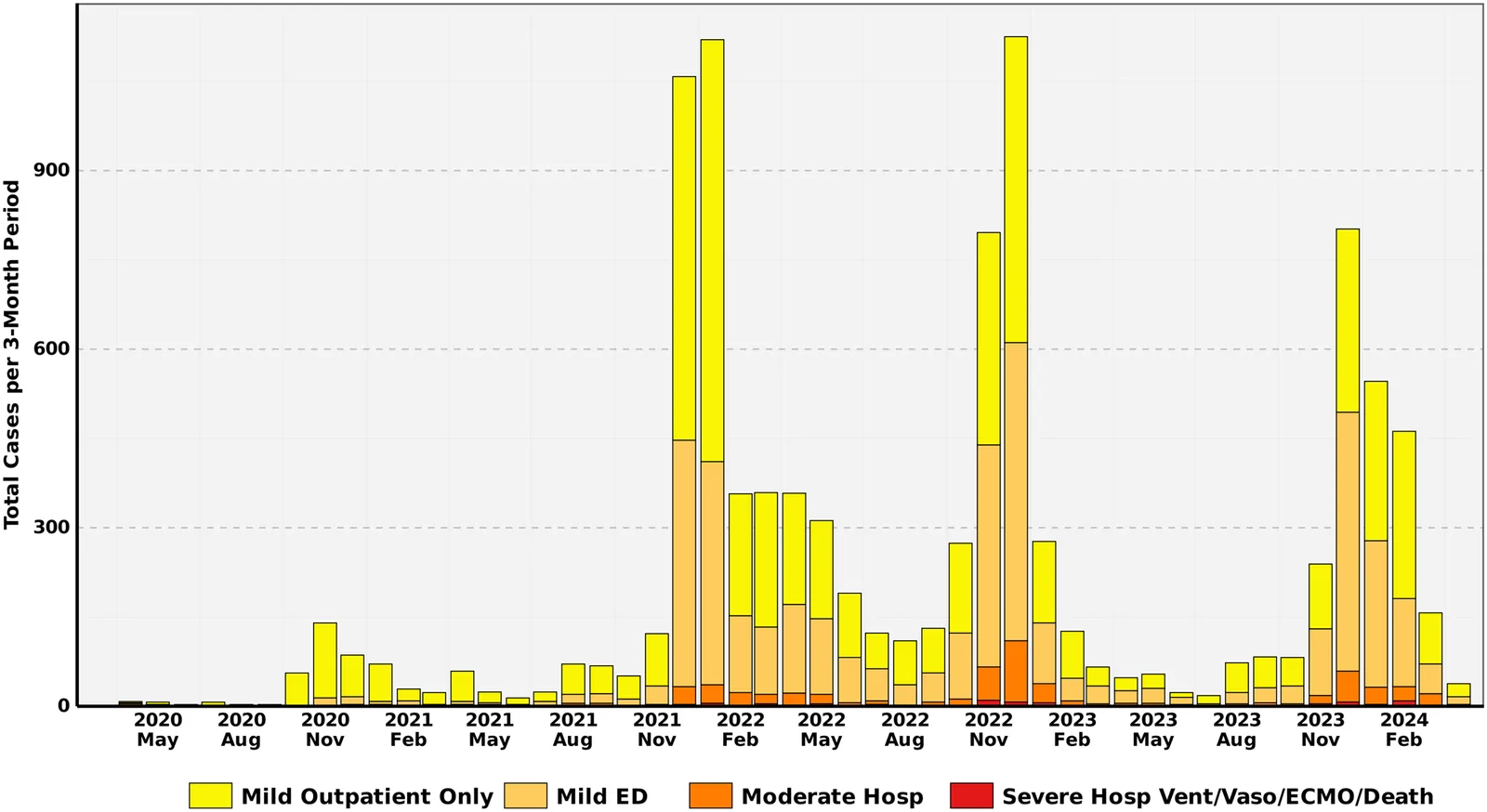
Monthly COVID-19 and influenza co-infection associated clinical outcomes, among patients aged ≤ 21 years, March 2020–April 2024. Figure 2. long description.

The final eligible population included 1,532,886 patients aged ≤21 years across 74 data partners in the N3C with SARS-CoV-2 or influenza from 1 March 2020 to 30 April 2024 (Figure 1). Of these, 1,208,609 (79%) had COVID-19-only, 312,715 (20%) had influenza only, and 11,562 (1%) met the co-infection definition (Supplementary eTable5). Overall, 61,278 (4%) hospitalizations were identified. Among co-infected patients, 6% (*n* = 705) had a hospitalization, compared with 4% (*n* = 48,698) among patients with COVID-19-only and 3.8% (*n* = 11,875) among those with influenza only. Three distinct waves of co-infection were observed: November 2021–March 2022, November 2022–March 2023, and November 2023–March 2024 (Figure 2). Frequencies by influenza season and SARS-CoV-2 variant predominance are shown in Supplementary eTable6.

After exclusions, analyses were conducted among 10,277 co-infected patients. The final selection risk ratio was null, indicating minimal evidence of selection bias (Supplementary eTable3). Most co-infected patients were aged 5–11 years (36%), male (52%), and White non-Hispanic (50%) (Table 1). White patients accounted for the largest proportion of co-infected patients (50%), followed by Black/African American (22%) and Hispanic or Latino patients (21%). The distribution was similar across phases, although the proportion of White patients was lower and Hispanic or Latino patients was higher in Phase 2. Twenty-seven per cent had at least one pre-existing diagnosis, and 13% had two or more; the most common conditions were obesity (24%), asthma (10%), and congenital or genetic disorders (5%). Among hospitalized co-infected patients, 0.8% of the overall cohort (13% of hospitalizations) met criteria for severe disease, defined as receipt of invasive mechanical ventilation, vasopressors/inotropes, extracorporeal membrane oxygenation, or death.

**Table 1.** Characteristics of patients aged ≤21 years with COVID-19 and influenza co-infection by period of COVID-19 pandemic phase^a^ – March 2020–April 2024 Table 1. long description.

Characteristic	No. of patients (%) with COVID-19 and influenza coinfection (*n* = 10,277)	No. of co-infected patients (%) during Phase 1 (*n* = 3,764)^b^	No. of co-infected patients (%) during Phase 2 (*n* = 6,513)^b^	Crude^c^ *p*-value
**Age group, yrs**				
0–4	1,922 (18.70%)	323 (8.6%)	1,599 (24.6%)	< 0.001
5–11	3,736 (36.35%)	1,398 (37.1%)	1,512 (23.2%)	
12–17	2,566 (24.97%)	1,054 (28.0%)	1,512 (23.2%)	
18–21	2,053 (19.98%)	989 (26.3%)	1,064 (16.3%)	
**Sex, *n* = 351 (3%) unknown**				
Male	5,304 (51.61%)	2,009 (53.4%)	3,295 (50.6%)	< 0.008
Female	4,973 (48.39%)	1,755 (46.6%)	3,218 (49.4%)	
**Race and ethnicity, *n* = 1,712 (14.8%) unknown** ^d^				
American Indian or Alaska Native	56 (0.54%)	NA	NA	0.01
Asian	341 (3.32%)	102 (2.7%)	239 (3.7%)	
Black/African American	2,289 (22.27%)	854 (22.7%)	1,435 (22.0%)	
Hispanic or Latino	2,185 (21.26%)	697 (18.5%)	1,488 (22.8%)	
Native Hawaiian or Other Pacific Islander	46 (0.45%)	NA	NA	
Other	263 (2.56%)	86 (2.3%)	177 (2.7%)	
White	5,097 (49.60%)	2,008 (53.3%)	3,089 (47.4%)	
**Any pre-existing diagnosis**				
None	6,247 (60.79%)	2,263 (60.1%)	3,984 (61.2%)	0.02
≥2 conditions	1,297 (12.62%)	439 (11.7%)	858 (13.2%)	
1 condition	2,733 (26.59%)	1,062 (28.2%)	1,671 (25.7%)	
**Specific pre-existing diagnoses** ^e,^ ^f^				
Asthma	1,066 (10.37%)	357 (9.5%)	709 (10.9%)	0.02
Cardiovascular	347 (3.38%)	112 (3.0%)	235 (3.6%)	0.06
Congenital/genetic	529 (5.15%)	172 (4.6%)	357 (5.5%)	0.03
Diabetes (type 1 or 2)	87 (0.85%)	32 (0.9%)	55 (0.8%)	0.93
Gastrointestinal	215 (2.09%)	69 (1.8%)	146 (2.2%)	0.11
Hematologic/immunologic	160 (1.56%)	53 (1.4%)	107 (1.6%)	0.24
Malignancy	109 (1.06%)	33 (0.9%)	76 (1.2%)	0.12
Metabolic	323 (3.14%)	108 (2.9%)	215 (3.3%)	0.16
Neonatal	146 (1.42%)	38 (1.0%)	108 (1.7%)	0.002
Neurologic/neuromuscular	449 (4.37%)	144 (3.8%)	305 (4.7%)	0.03
Obesity	2,416 (23.51%)	972 (25.8%)	1,444 (22.2%)	< 0.001
Renal	204 (1.99%)	72 (1.9%)	132 (2.0%)	0.58
Respiratory	265 (2.58%)	85 (2.3%)	180 (2.8%)	0.05
Technology dependent	139 (1.35%)	47 (1.2%)	92 (1.4%)	0.38
**Disease severity**				
Mild (outpatient visit only)	5,674 (55.21%)	2,443 (64.9%)	3,231 (49.6%)	< 0.001
Mild ED (outpatient with emergency department visit)	3,956 (38.49%)	1,172 (31.1%)	2,784 (42.7%)	
Moderate (hospitalized without requiring invasive ventilation, or ECMO)	563 (5.48%)	129 (3.4%)	434 (6.7%)	
Severe (hospitalized and requiring invasive ventilation, or ECMO, or death)	84 (0.82%)	20 (0.5%)	64 (1.0%)	

a Phase 1 was defined as 1 March 2020 to 31 March 2022, and Phase 2 included the period from 1 April 2022 to 30 April 2024.

b *N* = 1,285 patients excluded due to missing sex or race and ethnicity.

c Crude *p*-values reflect global comparisons of each multi-level variable across pandemic phases, calculated via univariate GEE models with a log link and clustering by data partner. These *p*-values indicate whether the overall distribution differs between pandemic phases, not individual row-level comparisons.

d Note that American Indian or Alaska Native and Native Hawaiian or Other Pacific Islander were omitted from COVID-19 Pandemic Phase breakdowns due to N3C data suppression rules (no cells <20 allowed) due to the potential for back calculation.

e Each specific pre-existing diagnosis was compared to the absence of such condition for statistical testing purposes.

f Note that patients with a history of transplant were omitted due to N3C data suppression rules (no cells <20 allowed) due to the potential for back calculation.

### Co-infection by pandemic phase

We found that 3,764 (37%) of co-infected patients were detected during Phase 1 of the COVID-19 pandemic, while 6,513 (63%) were detected during Phase 2, following the Omicron BA.1 wave and influenza resurgence (after 1 April 2022) (Table 1). Co-infected patients tended to be older during Phase 1 of the COVID-19 pandemic (54% age 12–21 years) and younger during Phase 2 (61% age 0–11 years), and this difference was statistically significant (*p* < 0.001). We did not detect a statistically significant difference in the burden of pre-existing diagnoses between pandemic phases (*p* = 0.02); however, we noted increases in cardiovascular, congenital/genetic, neonatal, and neuromuscular conditions, while obesity decreased. We observed an increase in disease severity from Phase 1 (4% inpatient) to Phase 2 (7% inpatient), *p* < 0.001. We observed similar changes among patients with COVID-19-only (3% vs. 6%; *p* < 0.001) and influenza-only (3% vs. 4%, *p* < 0.001) (Supplementary eTable7). The three waves of co-infection were primarily driven by those categorized as mild, followed by modest activity for those with moderate disease (Figure 2).

### Co-infection disease severity by age group and pre-existing diagnoses

Neither age group nor pre-existing diagnoses exhibited statistical interaction with pandemic phase; therefore, we did not stratify patients by pandemic phase. Overall, 6% (*n* = 647) of co-infected patients experienced Severe/Moderate disease (Table 2). Most co-infected patients with Severe/Moderate disease were age 0–11 years (69%). We also observed that co-infected patients with Severe/Moderate disease had a greater prevalence of two or more pre-existing diagnoses versus those who were Mild ED/outpatients (29% vs. 12%). Our final adjusted model following backward selection included age group and number of pre-existing diagnoses. We found that the adjusted risk of Severe/Moderate disease was 3.05 (95% CI: 2.35–3.97) times higher among patients aged 0–4 years, 1.86 (95% CI: 1.44–2.41) times higher among patients aged 5–11 years, and 1.50 (95% CI: 1.13–1.98) times higher among patients aged 12–17 years compared to the reference age group 18–21 years. The adjusted risk of Severe/Moderate disease was 2.89 (95% CI: 2.43–3.43) times higher among co-infected patients with two or more pre-existing diagnoses compared to the reference of no documented conditions. Patients with one pre-existing diagnosis had a modestly higher, but not statistically significant, adjusted risk of Severe/Moderate disease compared with patients with no documented conditions (aRR 1.19; 95% CI: 0.99–1.43).

**Table 2.** Modelled severe/moderate disease^a^ versus mild ED/outpatient disease^b^ associated with COVID-19 and influenza co-infection, patients aged ≤ 21 years, by age group and number of pre-existing diagnoses – National COVID Cohort Collaborative (N3C), March 1 2020–April 30 2024 (*n* = 10,277) Table 2. long description.

Variable	Severe/Moderate disease (*N* [%])	Mild ED/outpatient disease (*N* [%])	Risk ratio (95% CI)^c^
**Age group (yrs)**			
0–4	205 (31.7%)	1,717 (17.8%)	3.05 (2.35, 3.97)
5–11	241 (37.2%)	3,495 (36.3%)	1.86 (1.44, 2.41)
12–17	130 (20.1%)	2,436 (25.3%)	1.50 (1.13, 1.98)
18–21	71 (11.0%)	1,982 (20.6%)	[reference]
**Number of pre-existing diagnoses**			
≥2 conditions	186 (28.7%)	1,111 (11.5%)	2.89 (2.43, 3.43)
1 condition	158 (24.4%)	2,575 (26.7%)	1.19 (0.99, 1.43)
None	303 (46.8%)	5,944 (61.7%)	[reference]

a Severe/moderate disease is defined as hospitalized without critical care support, or hospitalized and requiring invasive ventilation, vasopressors/inotropes, or ECMO, or death.

b Mild ED/outpatient is defined as outpatient visit only or outpatient with emergency department visit.

c Risk ratios were compared using GEE models with a binomial family for categorical variables and a log link function. All models accounted for clustering by data partners and used backward selection (final model: age group and number of pre-existing diagnoses).

### Sensitivity analysis of co-infection case definition

We calculated the sensitivity, specificity, and PPV of patients in each co-infection case definition level (Supplementary eTable8). We note that our strictest case definition, COVID-19 and influenza positive laboratory result only, documented zero days apart, included 7,934 cases, whereas our broadest case definition, COVID-19 and influenza positive laboratory result or diagnosis, documented 14 days apart, included 10,691 cases. Overall, the sensitivity was 100% across all levels, specificity ranged from 99.8% to 99.9%, and PPV ranged from 76.1% to 87.5%. Finally, we repeated our calculation of the adjusted RR and 95% CIs of Severe/Moderate disease versus Mild ED/outpatient only across each case definition level (Supplementary eFigure1). For our chosen working case definition of COVID-19 and influenza positive laboratory results or diagnosis documented seven or fewer days apart, the specificity and PPV were 99.8% and 77.2%, respectively.

## Discussion

This N3C study developed and implemented a working EHR-based case definition for paediatric COVID-19 and influenza co-infection and found that approximately 6% of co-infected patients were hospitalized. These findings describe the clinical profile of co-infection after influenza activity returned to pre-pandemic levels. Our co-infection patient profile reflects what clinicians can expect in practice moving forward with endemic COVID-19 circulation in the United States. We found that the age distribution of co-infection was predominantly below age 12 years, which aligns with evidence that reported a greater burden of co-infection in younger children compared to adolescents [6]. We expected asthma and obesity to be common conditions among co-infected patients, as either can render patients more susceptible to respiratory infections and severe outcomes [33–36]. However, we could not analyse either separately in our GEE models due to a lack of statistical power. Our modelling indicated that co-infected patients aged 0–4 years had the highest calculated disease severity, which may be explained by infants younger than 6 months not being eligible for COVID-19 or influenza vaccination, among other factors [19, 37]. Importantly, the observed shift towards younger co-infected patients in Phase 2 may reflect a change in the underlying population composition rather than evidence that the effect of age on severity differed by pandemic phase. We did not observe a statistical interaction between age group and pandemic phase, suggesting that the association between younger age and Severe/Moderate disease remained consistent across phases.

The higher proportion of Severe/Moderate disease in Phase 2 may reflect changes in testing practices, patient characteristics, and respiratory virus circulation after relaxation of NPIs. While we expected this result from influenza, the result for COVID-19 warrants further consideration, as we expected greater severity during the Phase 1 prior to the availability of vaccines and therapeutics. This observed shift may be explained by three factors. First, the prevalence of pre-existing diagnoses increased during Phase 2. Second, our study examined case-based disease severity, and viral testing was more widespread during the first phase, meaning a broader spectrum of cases may have been detected compared to the second phase when milder or asymptomatic cases may have accessed at-home testing [38]. Third, younger patients may not have experienced other respiratory diseases due to NPIs (‘immunity debt’), and infections occurring following NPI relaxation may have greater pathogenicity and virulence [39, 40].

In the absence of a validated case definition for co-infection, our study implemented a practical EHR-based definition informed by prior studies [16, 17]. Based on our sensitivity analysis, we are confident that our co-infected population was less likely to be biased by including too many patients who were not actually co-infected. We interpreted the PPV as lower among patients using our case definition since co-infection is an uncommon condition, with a low prevalence.

These findings support consideration of early dual testing for SARS-CoV-2 and influenza during periods of co-circulation, particularly among younger children and patients with pre-existing diagnoses. Prompt identification of co-infection may help guide timely antiviral treatment, supportive care, and vaccination counselling for eligible patients and families. Most co-infected patients in this cohort were managed in outpatient or ED settings, although patients aged 0–4 years and those with multiple pre-existing diagnoses had higher risk for Severe/Moderate disease.

### Limitations

This study had several limitations. First, N3C data partners vary in terms of data collection practices and availability. We addressed this by including data partner as a clustering variable in our GEE models. Second, N3C facilities may not be generalizable to the rest of the US, and severe outcomes and pre-existing diagnoses may be overrepresented. Third, respiratory virus testing results from non-N3C facilities may be missed, resulting in underestimating mild co-infected cases and overestimating disease severity. Fourth, we were unable to capture intensive care unit (ICU) admissions as this variable is not uniformly reported in the N3C; however, we ensured we captured other indicators of intensive care (IMV, vasopressors/inotropes, ECMO). Although we grouped moderate and severe disease due to small numbers of severe cases, future studies should evaluate very severe outcomes separately, including IMV, ECMO, or death. Fifth, vaccination data in the N3C are incomplete, with no explicit indicator of non-vaccination; therefore, vaccination history was not considered and may represent an important unmeasured confounder. COVID-19 and influenza vaccination uptake varied over time and by age group during the study period, which may have influenced both the risk of co-infection and the likelihood of severe clinical outcomes. For example, differential vaccine eligibility and uptake by age may also have contributed to the observed shift towards younger age groups among co-infected patients in later phases. Additionally, vaccination may have reduced disease severity in later periods, potentially attenuating observed associations. Sixth, analyses by SARS-CoV-2 variant period and influenza season were precluded due to small sample sizes. Seventh, our definition of co-infection as SARS-CoV-2 and influenza detected within seven days was based on prior paediatric studies and reflects the overlapping incubation periods and clinical presentation of these respiratory viruses. However, this definition may include some sequential infections rather than true simultaneous co-infections, particularly in the context of delayed testing or prolonged viral shedding. To address this, we conducted sensitivity analyses using more restrictive and more inclusive temporal definitions, which demonstrated consistent findings, supporting the robustness of our results across reasonable case definitions. Eight, our N3C dataset did not include standardized symptom-level data; therefore, we could not characterize clinical presentation beyond healthcare utilization and disease severity. Although our case definitions included clinically diagnosed and laboratory-confirmed infections, we could not compare symptom profiles between these groups. Finally, given the nature of N3C EHR reporting, we assumed data were not missing at random and conducted a complete case analysis instead of imputation, potentially introducing selection bias. However, our calculated selection probabilities indicated that selection bias was unlikely to impact our results.

## Conclusions

This study leveraged an EHR data set of 1.5 million paediatric patients to develop and implement the largest cohort of paediatric COVID-19 and influenza co-infections to date. We provided valuable results on the profile of co-infection during a time when influenza returned to pre-pandemic levels. Patients with co-infection tend to be younger and have pre-existing diagnoses. Our sensitivity analysis supported our case definition of co-infection as COVID-19 and influenza laboratory result or diagnosis seven or fewer days apart, which can be used in future studies. Co-infections should be considered by paediatricians from November through March during respiratory virus seasons. Further analytic studies are needed to compare the risk of severe clinical outcomes between co-infection and single infection, explore differences in human biological responses, and clarify the transmission dynamics of both diseases at the population level.

## Supporting information

10.1017/S0950268826101915.sm001 Naqvi et al. supplementary material Naqvi et al. supplementary material

## Data Availability

The code for this study (DUR: RP-0585B0) is located within the N3C Data Enclave and can be accessed upon request, provided the user already has access to the N3C.

## Supplementary material

The supplementary material for this article can be found at http://doi.org/10.1017/S0950268826101915.

## N3C attribution

The analyses described in this publication were conducted with data or tools accessed through the National Center for Advancing Translational Sciences (NCATS) N3C Data Enclave https://covid.cd2h.org and N3C Attribution & Publication Policy v 1.2-2020-08-25b supported by NCATS Contract No. 75N95023D00001, Axle Informatics Subcontract: NCATS-P00438-B. This research was possible because of the patients whose information is included within the data and the organizations (https://ncats.nih.gov/n3c/resources/data-contribution/data-transfer-agreement-signatories) and scientists who have contributed to the on-going development of this community resource [https://doi.org/10.1093/jamia/ocaa196].

## Data partners with released data

The following institutions whose data are released or pending:

Available: Advocate Health Care Network – UL1TR002389: The Institute for Translational Medicine (ITM) • Aurora Health Care Inc. – UL1TR002373: Wisconsin Network For Health Research • Boston University Medical Campus – UL1TR001430: Boston University Clinical and Translational Science Institute • Brown University – U54GM115677: Advance Clinical Translational Research (Advance-CTR) • Carilion Clinic – UL1TR003015: iTHRIV Integrated Translational health Research Institute of Virginia • Case Western Reserve University – UL1TR002548: The Clinical & Translational Science Collaborative of Cleveland (CTSC) • Charleston Area Medical Center – U54GM104942: West Virginia Clinical and Translational Science Institute (WVCTSI) • Children’s Hospital Colorado – UL1TR002535: Colorado Clinical and Translational Sciences Institute • Columbia University Irving Medical Center – UL1TR001873: Irving Institute for Clinical and Translational Research • Dartmouth College – None (Voluntary) Duke University – UL1TR002553: Duke Clinical and Translational Science Institute • George Washington Children’s Research Institute – UL1TR001876: Clinical and Translational Science Institute at Children’s National (CTSA-CN) • George Washington University – UL1TR001876: Clinical and Translational Science Institute at Children’s National (CTSA-CN) • Harvard Medical School – UL1TR002541: Harvard Catalyst • Indiana University School of Medicine – UL1TR002529: Indiana Clinical and Translational Science Institute • Johns Hopkins University – UL1TR003098: Johns Hopkins Institute for Clinical and Translational Research • Louisiana Public Health Institute – None (Voluntary) • Loyola Medicine – Loyola University Medical Center • Loyola University Medical Center – UL1TR002389: The Institute for Translational Medicine (ITM) • Maine Medical Center – U54GM115516: Northern New England Clinical & Translational Research (NNE-CTR) Network • Mary Hitchcock Memorial Hospital & Dartmouth Hitchcock Clinic – None (Voluntary) • Massachusetts General Brigham – UL1TR002541: Harvard Catalyst • Mayo Clinic Rochester – UL1TR002377: Mayo Clinic Center for Clinical and Translational Science (CCaTS) • Medical University of South Carolina – UL1TR001450: South Carolina Clinical & Translational Research Institute (SCTR) • MITRE Corporation – None (Voluntary) • Montefiore Medical Center – UL1TR002556: Institute for Clinical and Translational Research at Einstein and Montefiore • Nemours – U54GM104941: Delaware CTR ACCEL Program • NorthShore University HealthSystem – UL1TR002389: The Institute for Translational Medicine (ITM) • Northwestern University at Chicago – UL1TR001422: Northwestern University Clinical and Translational Science Institute (NUCATS) • OCHIN – INV-018455: Bill and Melinda Gates Foundation grant to Sage Bionetworks • Oregon Health & Science University – UL1TR002369: Oregon Clinical and Translational Research Institute • Penn State Health Milton S. Hershey Medical Center – UL1TR002014: Penn State Clinical and Translational Science Institute • Rush University Medical Center – UL1TR002389: The Institute for Translational Medicine (ITM) • Rutgers, The State University of New Jersey – UL1TR003017: New Jersey Alliance for Clinical and Translational Science • Stony Brook University – U24TR002306 • The Alliance at the University of Puerto Rico, Medical Sciences Campus – U54GM133807: Hispanic Alliance for Clinical and Translational Research (The Alliance) • The Ohio State University – UL1TR002733: Center for Clinical and Translational Science • The State University of New York at Buffalo – UL1TR001412: Clinical and Translational Science Institute • The University of Chicago – UL1TR002389: The Institute for Translational Medicine (ITM) • The University of Iowa – UL1TR002537: Institute for Clinical and Translational Science • The University of Miami Leonard M. Miller School of Medicine – UL1TR002736: University of Miami Clinical and Translational Science Institute • The University of Michigan at Ann Arbor – UL1TR002240: Michigan Institute for Clinical and Health Research • The University of Texas Health Science Center at Houston – UL1TR003167: Center for Clinical and Translational Sciences (CCTS) • The University of Texas Medical Branch at Galveston – UL1TR001439: The Institute for Translational Sciences • The University of Utah – UL1TR002538: Uhealth Center for Clinical and Translational Science • Tufts Medical Center – UL1TR002544: Tufts Clinical and Translational Science Institute • Tulane University – UL1TR003096: Center for Clinical and Translational Science • The Queens Medical Center – None (Voluntary) • University Medical Center New Orleans – U54GM104940: Louisiana Clinical and Translational Science (LA CaTS) Center • University of Alabama at Birmingham – UL1TR003096: Center for Clinical and Translational Science • University of Arkansas for Medical Sciences – UL1TR003107: UAMS Translational Research Institute • University of Cincinnati – UL1TR001425: Center for Clinical and Translational Science and Training • University of Colorado Denver, Anschutz Medical Campus – UL1TR002535: Colorado Clinical and Translational Sciences Institute • University of Illinois at Chicago – UL1TR002003: UIC Center for Clinical and Translational Science • University of Kansas Medical Center – UL1TR002366: Frontiers: University of Kansas Clinical and Translational Science Institute • University of Kentucky – UL1TR001998: UK Center for Clinical and Translational Science • University of Massachusetts Medical School Worcester – UL1TR001453: The UMass Center for Clinical and Translational Science (UMCCTS) • University Medical Center of Southern Nevada – None (voluntary) • University of Minnesota – UL1TR002494: Clinical and Translational Science Institute • University of Mississippi Medical Center – U54GM115428: Mississippi Center for Clinical and Translational Research (CCTR) • University of Nebraska Medical Center – U54GM115458: Great Plains IDeA-Clinical & Translational Research • University of North Carolina at Chapel Hill – UL1TR002489: North Carolina Translational and Clinical Science Institute • University of Oklahoma Health Sciences Center – U54GM104938: Oklahoma Clinical and Translational Science Institute (OCTSI) • University of Pittsburgh – UL1TR001857: The Clinical and Translational Science Institute (CTSI) • University of Pennsylvania – UL1TR001878: Institute for Translational Medicine and Therapeutics • University of Rochester – UL1TR002001: UR Clinical & Translational Science Institute • University of Southern California – UL1TR001855: The Southern California Clinical and Translational Science Institute (SC CTSI) • University of Vermont – U54GM115516: Northern New England Clinical & Translational Research (NNE-CTR) Network • University of Virginia – UL1TR003015: iTHRIV Integrated Translational health Research Institute of Virginia • University of Washington – UL1TR002319: Institute of Translational Health Sciences • University of Wisconsin-Madison – UL1TR002373: UW Institute for Clinical and Translational Research • Vanderbilt University Medical Center – UL1TR002243: Vanderbilt Institute for Clinical and Translational Research • Virginia Commonwealth University – UL1TR002649: C. Kenneth and Dianne Wright Center for Clinical and Translational Research • Wake Forest University Health Sciences – UL1TR001420: Wake Forest Clinical and Translational Science Institute • Washington University in St. Louis – UL1TR002345: Institute of Clinical and Translational Sciences • Weill Medical College of Cornell University – UL1TR002384: Weill Cornell Medicine Clinical and Translational Science Center • West Virginia University – U54GM104942: West Virginia Clinical and Translational Science Institute (WVCTSI) Submitted: Icahn School of Medicine at Mount Sinai – UL1TR001433: ConduITS Institute for Translational Sciences • The University of Texas Health Science Center at Tyler – UL1TR003167: Center for Clinical and Translational Sciences (CCTS) • University of California, Davis – UL1TR001860: UCDavis Health Clinical and Translational Science Center • University of California, Irvine – UL1TR001414: The UC Irvine Institute for Clinical and Translational Science (ICTS) • University of California, Los Angeles – UL1TR001881: UCLA Clinical Translational Science Institute • University of California, San Diego – UL1TR001442: Altman Clinical and Translational Research Institute • University of California, San Francisco – UL1TR001872: UCSF Clinical and Translational Science Institute NYU Langone Health Clinical Science Core, Data Resource Core, and PASC Biorepository Core – OTA-21-015A: Post-Acute Sequelae of SARS-CoV-2 Infection Initiative (RECOVER) Pending: Arkansas Children’s Hospital – UL1TR003107: UAMS Translational Research Institute • Baylor College of Medicine – None (Voluntary) • Children’s Hospital of Philadelphia – UL1TR001878: Institute for Translational Medicine and Therapeutics • Cincinnati Children’s Hospital Medical Center – UL1TR001425: Center for Clinical and Translational Science and Training • Emory University – UL1TR002378: Georgia Clinical and Translational Science Alliance • HonorHealth – None (Voluntary) • Loyola University Chicago – UL1TR002389: The Institute for Translational Medicine (ITM) • Medical College of Wisconsin – UL1TR001436: Clinical and Translational Science Institute of Southeast Wisconsin • MedStar Health Research Institute – None (Voluntary) • Georgetown University – UL1TR001409: The Georgetown-Howard Universities Center for Clinical and Translational Science (GHUCCTS) • MetroHealth – None (Voluntary) • Montana State University – U54GM115371: American Indian/Alaska Native CTR • NYU Langone Medical Center – UL1TR001445: Langone Health’s Clinical and Translational Science Institute • Ochsner Medical Center – U54GM104940: Louisiana Clinical and Translational Science (LA CaTS) Center • Regenstrief Institute – UL1TR002529: Indiana Clinical and Translational Science Institute • Sanford Research – None (Voluntary) • Stanford University – UL1TR003142: Spectrum: The Stanford Center for Clinical and Translational Research and Education • The Rockefeller University – UL1TR001866: Center for Clinical and Translational Science • The Scripps Research Institute – UL1TR002550: Scripps Research Translational Institute • University of Florida – UL1TR001427: UF Clinical and Translational Science Institute • University of New Mexico Health Sciences Center – UL1TR001449: University of New Mexico Clinical and Translational Science Center • University of Texas Health Science Center at San Antonio – UL1TR002645: Institute for Integration of Medicine and Science • Yale New Haven Hospital – UL1TR001863: Yale Center for Clinical Investigation.

## Long descriptions

### Figure 1 Long description

The flowchart follows this sequence:

* Top box: N 3 C Data Enclave unique patients n = 22,777,625.

* First exclusion step: Two dashed boxes branch out. Left: Excluded patients with missing age n = 78,218. Right: Excluded adults older than age 21 n = 18,518,130.

* Second main box: N 3 C Enclave unique patients less than or equal to 21 n = 4,181,277.

* Second exclusion step: Dashed box to the right: Excluded patients less than or equal to 21 without evidence of S A R S-C o V-2 or influenza virus n = 2,482,860.

* Third main box: Total patient cohort n = 1,698,417.

* Third exclusion step: Dashed box to the left: Data partner level exclusions from statistical analyses super 1: n = 12 partners (165,531 unique patients).

* Horizontal branching: The flow splits into three categories:

1. Total patients with C O V I D-19 n = 1,208,609.

2. Total patients with co-infection n = 11,562.

3. Total patients with influenza n = 312,715.

* Final exclusion step: From the co-infection branch, a dashed box to the left: Patient level exclusions from statistical analyses super 2: Sex n = 351, Race/ethnicity n = 985.

* Bottom box: Final effective sample size for analysis n = 10,277.

Navigate back to [Figure 1].

### Figure 2 Long description

The X axis represents time in 3 month intervals from May 2020 to February 2024. The Y axis represents Total Cases per 3 Month Period, ranging from 0 to 900 with increments of 300.

Each bar is stacked with four color coded categories from bottom to top.

1. Severe Hosp Vent or Vaso or E C M O or Death (Red): The smallest segment, appearing as a thin line at the base of some bars.

2. Moderate Hosp (Orange): A small segment above severe cases.

3. Mild E D (Light Orange): A significant middle segment representing emergency department visits.

4. Mild Outpatient Only (Yellow): The largest segment forming the top of most bars.

The data shows three distinct seasonal peaks.

- The first major peak occurs between November 2021 and February 2022, reaching over 1,100 total cases.

- The second major peak occurs around November 2022, reaching approximately 1,150 total cases.

- A third, slightly lower peak occurs around November 2023, reaching approximately 800 total cases.

- Between these peaks, case counts drop significantly, often falling below 100 cases during the summer months (May through August).

Navigate back to [Figure 2].

### [Table 1] Long description

The table compares patient characteristics between Phase 1 (March 2020 to March 2022) and Phase 2 (April 2022 to April 2024).

* Age group: The 5 to 11 age group was the largest overall (36.35 percent). Phase 2 saw a significant increase in the 0 to 4 age group (24.6 percent) compared to Phase 1 (8.6 percent). p-value is less than 0.001.

* Sex: Males accounted for 51.61 percent and females 48.39 percent of the total. p-value is less than 0.008.

* Race and ethnicity: White patients were the largest group (49.60 percent), followed by Black or African American (22.27 percent) and Hispanic or Latino (21.26 percent). p-value is 0.01.

* Pre-existing conditions: 60.79 percent of patients had no pre-existing diagnosis. Obesity was the most common specific diagnosis (23.51 percent), followed by Asthma (10.37 percent). Obesity prevalence was higher in Phase 1 (25.8 percent) than Phase 2 (22.2 percent). p-value for obesity is less than 0.001.

* Disease severity: Most cases were mild (55.21 percent outpatient only; 38.49 percent with E D visit). Phase 2 showed an increase in moderate (6.7 percent) and severe (1.0 percent) cases compared to Phase 1 (3.4 percent and 0.5 percent respectively). p-value is less than 0.001.

Navigate back to [Table 1].

### Table 2 Long description

The table contains four columns: Variable, Severe or Moderate disease (N and percentage), Mild E D or outpatient disease (N and percentage), and Risk ratio with 95 percent C I.

Under Age group in years:

- 0 to 4: 205 (31.7 percent) severe, 1,717 (17.8 percent) mild, risk ratio 3.05 (2.35, 3.97).

- 5 to 11: 241 (37.2 percent) severe, 3,495 (36.3 percent) mild, risk ratio 1.86 (1.44, 2.41).

- 12 to 17: 130 (20.1 percent) severe, 2,436 (25.3 percent) mild, risk ratio 1.50 (1.13, 1.98).

- 18 to 21: 71 (11.0 percent) severe, 1,982 (20.6 percent) mild, serving as the reference group.

Under Number of pre-existing diagnoses:

- 2 or more conditions: 186 (28.7 percent) severe, 1,111 (11.5 percent) mild, risk ratio 2.89 (2.43, 3.43).

- 1 condition: 158 (24.4 percent) severe, 2,575 (26.7 percent) mild, risk ratio 1.19 (0.99, 1.43).

- None: 303 (46.8 percent) severe, 5,944 (61.7 percent) mild, serving as the reference group.

Navigate back to [Table 2].
