# Epidemiological characteristics of paediatric COVID-19 and influenza co-infections in the United States, 2020–2024 – CORRIGENDUM

**DOI:** 10.1017/S0950268826101988

**Published:** 2026-09-04

**Authors:** Ozair H. Naqvi, Aaron M. Wendelboe, William H. Beasley, Donna L. Tyungu, Kai Ding, Katrin G. Kuhn, James J. Cheng, A. Jerrod Anzalone, Amanda E. Janitz

When this article was published in *Epidemiology and Infection* it included an error in the name of the author A. Jerrod Anzalone. This has been corrected online and in the article.

The authors apologise for this error.
