# Supplementary material for: Epidemiological characteristics of paediatric COVID-19 and influenza co-infections in the United States, 2020–2024
Source: Epidemiol Infect. 2026 Jul 29;154:e105. doi: 10.1017/S0950268826101915 (PMC13437991; doi:10.1017/S0950268826101915)
Supplement: Supplementary file 1 [file S0950268826101915sup001.docx]

**SUPPORTING INFORMATION**

**Epidemiological characteristics of paediatric COVID-19 and influenza co-infections in the United States, 2020–2024**

Ozair H. Naqvi, Ph.D..; Aaron M. Wendelboe, Ph.D.; William H. Beasley, Ph.D.; Donna L. Tyungu, M.D.; Kai Ding, Ph.D.; Katrin G. Kuhn, Ph.D.; James J. Cheng, M.A.; A. Jerrod Anzalone, Ph.D.; Amanda E. Janitz, Ph.D.; on behalf of N3C consortium

**eMethods.** Detailed Methods

**eTable1.** N3C Data Partner Inclusion/exclusion Criteria

**eTable2.** Characteristics of patients aged ≤ 21 years with COVID-19 and influenza co-infection, included and excluded from analysis—March 2020–April 2024.

**eTable3.** Assessment of selection bias (using a cohort study framework) in excluding patients with unknown sex or race and ethnicity

**eTable4.** Full List of Variables and Concept Set Definitions Used to Form Final Patient Cohort

**eTable5.** Characteristics of patients aged ≤ 21 years with COVID-19, influenza, and co-infection,—National Clinical Cohort Collaborative (N3C), March 2020–April 2024

**eTable6.** Frequency and proportion of patients aged ≤ 21 years with COVID-19 and influenza co-infection, by influenza season and SARS-CoV-2 variant period of predominance—National Clinical Cohort Collaborative (N3C), March 2020– April 2024

**eTable7.** Frequency and proportion of clinical outcomes among patients aged ≤ 21 years with COVID-19 and influenza, by COVID-19 pandemic phase—National Clinical Cohort Collaborative (N3C), March 2020– April 2024

**eTable8.**Sensitivity, specificity, and positive predictive value of varying COVID-19 and influenza co-infection case definitions among patients aged < 21 years—National Clinical Cohort Collaborative (N3C), March 2020–April 2024.

**eFigure1.** Sensitivity analysis of modelled Severe/Moderate disease versus mild ED/outpatient disease associated with COVID-19 and influenza co-infection, patients aged ≤ 21 years, by age group and number of pre-existing diagnoses, by co-infection case definition—National Clinical Cohort Collaborative (N3C), March 2020–April 2024

**eMethods.** Detailed Methods

Study Design:

A retrospective observational study of patients 21 years and younger in the N3C was performed to assess the overall burden and clinical outcomes associated with COVID-19 and influenza co-infection from March 2020–April 2024. We followed the Enhancing the Quality and Transparency of Health Research (EQUATOR) reporting, Reporting of Studies Conducted Using Observational Routinely Collected Health Data (RECORD) guidelines [1].

Setting and Study Time Frame:

The N3C Data Enclave collects and harmonizes longitudinal EHR data (with a 2-year “lookback” period to 1 January 2018 to capture information about pre-existing health conditions for patients) on all persons with a positive SARS-CoV-2 laboratory test [polymerase chain reaction (PCR), antigen (Ag)] or a COVID-19 diagnostic code [2].

We analysed N3C data on patients aged 21 years and younger across 81 initial data partners in the N3C from 1 March 2020–30 April 2024 (n = 1,698,417). We chose 21 years and younger to include adolescents and young adults given the physiological changes such persons experience during this phase of life, in addition to ensuring those with complex chronic conditions are well represented [3]. We extracted all data on 1 June 2024 (N3C release version 169) using the Observational Medical Outcomes Partnership (OMOP) Common Data Model [4]. All clinical concept definitions were created in the N3C Enclave, with input from at least one clinician and one informatician, both with subject matter expertise in the relevant domains [5, 6].

Study Population:

The N3C defined patients with COVID-19 based on two criteria: 1) SARS-CoV-2 testing (LOINC codes) and 2) diagnostic condition codes (ICD-10-CM and SNOMED CT). We excluded patients with only a positive SARS-CoV-2 antibody test as they do not reliably indicate the timing of acute infection [7]. N3C also provides data on non-COVID-19 cases who are matched (2:1 non-COVID-19: COVID-19) to COVID-19 cases based on data partner, age, sex, race, and ethnicity. Patients with influenza were also identified using LOINC, ICD-10-CM and SNOMED CT codes [8]. To ensure diagnostic accuracy, we incorporated ICD-10-CM codes validated by Antoon et al. (2024) [9]. We excluded patients with a positive influenza antibody test or diagnostic codes for influenza subtypes other than A or B. We restricted our analysis to patients with influenza A or B as these are the predominant seasonal influenza viruses responsible for acute illness and are most reliably captured in administrative and laboratory coding systems. We did not restrict inclusion based on symptom status; therefore, asymptomatic or screening-identified cases may have been included.

We used the N3C definition of index dates, defined as a patient’s first encounter involving COVID-19 (laboratory result or diagnostic code) [8]. We applied this same definition to the influenza group. We defined co-infection as a patient with an index date for COVID-19 and influenza seven or fewer days apart. This definition was based on similar studies of paediatric co-infections involving COVID-19 [10, 11]. For patients with multiple qualifying encounters or test results, the first documented COVID-19 index date and first documented influenza index date were used; subsequent encounters were used only to classify associated clinical outcomes within the defined outcome window. Patients identified in the co-infection group were not included in the COVID-19-only or influenza-only groups. We excluded sites that did not report minimal data elements from our study (n = 12) and conducted a complete case analysis; therefore, individuals with unknown values for variables of interest were excluded from statistical testing (n = 1,285, see **eTable2** for excluded patient characteristics). We assessed our exclusion of co-infected patients using selection probabilities based on guidance from Lash et al. (2009) [12]. In **eTable3**, α, β, γ, and δ represent the proportions of severe and moderate/mild cases included (e.g., α = A°/A), and the selection odds ratios (rE = α/β for co-infection, rnE = γ/δ for infection alone) capture differential inclusion by severity. The final equation estimates the bias in the effect estimate due to differential selection, showing minimal effect (bias = 0).

Variables:

*Patient Measurements*

We incorporated patient age group (at infection) based on CDC’s criteria used to assess infection-associated hospitalizations among children, adolescents, and young adults (0 to 4 years, 5 to 11 years, 12 to 17 years, or 18 to 21 years) [13]. We also categorized sex (male or female) and race/ethnicity (American Indian/Alaska Native, Asian, Black/African American, Hispanic or Latino, Native Hawaiian or Other Pacific Islander, Other, and White). We defined and assigned pre-existing diagnoses to patients using the Pediatric Complex Chronic Conditions (PCCC) [14] and certain comorbidities in the N3C [(asthma [15], obesity [16, 17], and diabetes mellitus (types 1 or 2)] [18] (**eTable4**) [18, 19]. If a patient had no documentation of a pre-existing diagnosis, the corresponding variable would be indicated as “no”. To assess the impact of medical complexity on severe outcomes, we calculated the frequency and percentage of patients with one, two or more, or no pre-existing diagnoses documented. We defined obesity based on Centers for Disease Control and Prevention (CDC) Growth Charts of Children 2-20 years [20], where a patient was considered obese if their body mass index (BMI) was in the ≥ 95th percentile for their age and sex (persons age 21 years were categorized using a BMI ≥ 30) [16, 21]. If a patient had no documentation of a pre-existing diagnosis, the corresponding variable would be indicated as “no”.

We defined two pandemic phases (Phase 1 and 2), based on the period before and after influenza reemerged in 2022 [22]. Phase 1 was defined as 1 March 2020 to 31 March 2022, which included the initial emergence of the Omicron strain. Phase 2 included the period from 1 April 2022 to 30 April 2024 which was largely been dominated by subvariants of the Omicron strain [23]. We were also interested in assessing the frequency and proportion of co-infection detections by influenza season and SARS-CoV-2 variant period of predominance. We defined influenza seasons as 1 August to 31 July of the following year, and defined SARS-CoV-2 period of predominance as the following [23]:

- Wild-Type: 1 March 2020–30 July 2021
- Delta: 1 August 2021–18 December 2021
- Omicron-BA: 19 December 2021–28 January 2023
- Omicron-XBB: 29 January 2023–6 January 2024
- Omicron-JN.1: 7 January 2024–30 April 2024

*Clinical Outcomes*

We defined COVID-19-associated patient encounters using logic employed by the N3C which were adapted to align with CDC guidelines [24]. We defined a COVID-19-associated emergency department (ED) visit as a patient who had an ED record with COVID-19 documented in the EHR rom 14 days before to 14 days after the index date (Mild ED). We defined a COVID-19-associated hospitalization in the same way. We also used the same criteria for influenza-related outcomes, in line with CDC guidelines [25]. If a patient was identified in both the ED and hospital, they would be included in the hospitalization category (Moderate disease). Hospitalised patients were flagged for severe disease if they had any of the following during their visit: use of vasopressors/inotropes, IMV, extracorporeal membrane oxygenation (ECMO), or death (inpatient death or discharge to hospice) recorded in the 60 days following the index date. Patients with no recorded ED or hospitalization visit were categorized as outpatients.

We assigned disease severity progression as the following using an adapted version of the Clinical Progression Scale (CPS) established by the World Health Organization (WHO) and used in prior N3C paediatric studies [26]:

- Mild (outpatient only)
- Mild ED (outpatient with ED visit)
- Moderate (hospitalised without requiring IMV, vasopressors/inotropes, or ECMO)
- Severe (hospitalised and requiring IMV, vasopressors/inotropes, ECMO, or death)

Statistical Methods:

We restricted patients to those with co-infections to investigate the change in demographics, pre-existing diagnoses, and clinical outcomes by pandemic phase. We calculated descriptive statistics using frequencies and proportions for patients by age group, sex, race and ethnicity, pre-existing diagnoses, and clinical outcomes. We then investigated the relationship between age group, number of pre-existing diagnoses, and clinical outcomes. We conducted statistical testing using generalised estimating equations (GEE) with a binomial distribution and log link function to calculate the risk ratios (RR) and 95% confidence intervals (CI) of each variable of interest using the geepack package in R [27]. All models used data partners as a clustering variable to account for differences in N3C site reporting. We modelled Severe/Moderate disease versus Mild ED/Mild outpatient only (reference group). We also assessed statistical interaction between each exposure variable and pandemic phase. If an interaction was detected (p < 0.05), we would stratify models by pandemic phase. To evaluate confounding, we used a change-in-estimate approach implemented through backward elimination. Candidate covariates were sequentially removed from the multivariable model and retained if their removal changed the primary exposure parameter estimate by >10%.

Finally, we conducted two sensitivity analyses. First, we compared the sensitivity, specificity, and positive predictive value of our co-infection case definition compared against a gold standard (COVID-19 and influenza positive laboratory results documented on the same day). For consistency, we conducted the same exclusions of data partner and missing sex or race and ethnicity for each level of case definition as the main analysis. We used the following definitions of co-infection as comparisons (with sample sizes in **eTable8**):

- COVID-19 and influenza positive laboratory result only, documented *zero* days apart (Gold Standard)
- COVID-19 and influenza diagnosis or laboratory result, documented *zero* days apart
- COVID-19 and influenza diagnosis or laboratory result, documented *three* days apart
- COVID-19 and influenza diagnosis or laboratory result, documented *seven* days apart (our default case definition)
- COVID-19 and influenza diagnosis or laboratory result, documented *ten* days apart
- COVID-19 and influenza diagnosis or laboratory result, documented *fourteen* days apart

Second, we explored how each case definition impacted our adjusted risk of Severe/Moderate disease (**eFigure1**). Similar to the first sensitivity analysis, for consistency, we maintained sex, race and ethnicity, and data partner exclusions based on our primary analysis.

We used an alpha level of 0.05 for all statistical testing. All analyses were performed within the N3C Data Enclave using R v4.1 in accordance with N3C privacy and download review policies [4]. This retrospective observational study received Institutional Review Board (IRB) approval from the University of Oklahoma Health Sciences (#16868) and is housed in a centralized IRB at Johns Hopkins University (#00249128). The N3C Data Access Committee reviewed and approved the limited dataset used for this study (DUR RP-0585B0).

**eTable1.** N3C Data Partner Inclusion/exclusion Criteria^1 2^

| **Data Partner** | **Data on paediatric patients** | **Data on paediatric COVID patients** | **Data on paediatric influenza patients** | **Data on COVID-19 ED and hospitalised patients** | **Data on influenza ED and hospitalised patients** |
| --- | --- | --- | --- | --- | --- |
| **A** |  | **–** | **–** | **–** | **–** |
| **B** |  |  |  |  | **–** |
| **C** |  |  |  |  |  |

1. In this example, Data Partners A and B would be excluded from the analysis.
2. Full list of data partners omitted due to N3C data privacy rules.

**eTable2.** Characteristics of patients aged ≤ 21 years with COVID-19 and influenza co-infection, included and excluded from analysis—March 2020–April 2024.

| **Characteristic** | **No. of patients (%) with COVID-19 and influenza co-infection included (n = 10,277)** | **No. of patients (%) with COVID-19 and influenza co-infection excluded (n = 1,285)** |
| --- | --- | --- |
| **Age group, yrs** |  |  |
| 0-4 | 1,922 (18.70%) | 302 (23.50%) |
| 5-11 | 3,736 (36.35%) | 468 (36.42%) |
| 12-17 | 2,566 (24.97%) | 276 (21.48%) |
| 18-21 | 2,053 (19.98%) | 239 (18.60%) |
| **Sex, n = 351 (3%) unknown** | | |
| Male | 5,304 (51.61%) | 488 (37.98%) |
| Female | 4,973 (48.39%) | 446 (34.71%) |
| **Race and ethnicity, n = 985 (9%) unknown^1^** | | |
| American Indian or Alaska Native | 56 (0.54%) | NA |
| Asian | 341 (3.32%) | 44 (3.42%) |
| Black/African American | 2,289 (22.27%) | 95 (7.93%) |
| Hispanic or Latino | 2,185 (21.26%) | NA |
| Native Hawaiian or Other Pacific Islander | 46 (0.45%) | NA |
| Other | 263 (2.56%) | NA |
| White | 5,097 (49.60%) | 156 (12.14%) |
| **Any pre-existing diagnosis** |  |  |
| None | 6,247 (60.79%) | 912 (70.97%) |
| ≥2 conditions | 1,297 (12.62%) | 96 (7.47%) |
| 1 condition | 2,733 (26.59%) | 277 (21.56%) |
| **Specific pre-existing diagnoses^2 3^** | | |
| Asthma | 1,066 (10.37%) | 239 (18.60%) |
| Cardiovascular | 347 (3.38%) | 30 (2.33%) |
| Congenital/Genetic | 529 (5.15%) | 58 (4.51%) |
| Diabetes (Type 1 or 2) | 87 (0.85%) | NA |
| Gastrointestinal | 215 (2.09%) | NA |
| Hematologic/Immunologic | 160 (1.56%) | NA |
| Malignancy | 109 (1.06%) | NA |
| Metabolic | 323 (3.14%) | 31 (2.41%) |
| Neonatal | 146 (1.42%) | NA |
| Neurologic/Neuromuscular | 449 (4.37%) | 39 (3.04%) |
| Obesity | 2,416 (23.51%) | 158 (12.30%) |
| Renal | 204 (1.99%) | 20 (1.56%) |
| Respiratory | 265 (2.58%) | 25 (1.95%) |
| Technology Dependent | 139 (1.35%) | NA |
| **Disease severity^4^** |  |  |
| Mild (outpatient visit only) | 5,674 (55.21%) | 811 (63.11%) |
| Mild ED (outpatient with emergency department visit) | 3,956 (38.49%) | 416 (32.37%) |
| Moderate (hospitalised without requiring invasive ventilation, or ECMO) | 563 (5.48%) | NA |
| Severe (hospitalised and requiring invasive ventilation, or ECMO, or death) | 84 (0.82%) | NA |

1. Note that American Indian or Alaska Native, Hispanic or Latino, Native Hawaiian or Other Pacific Islander, and Other were omitted due to N3C data suppression rules (no cells < 20 allowed).
2. Note that Diabetes (Type 1 or 2), Gastrointestinal, Hematologic/Immunologic, Malignancy, Neonatal, Technology Dependent were omitted due to N3C data suppression rules (no cells < 20 allowed).
3. Note that patients with a history of transplant were omitted due to N3C data suppression rules (no cells < 20 allowed) due to the potential for back calculation.
4. Note that disease severity categories Moderate (hospitalised without requiring invasive ventilation, or ECMO) and Severe (hospitalised and requiring invasive ventilation, or ECMO, or death) were omitted due to N3C data suppression rules (no cells < 20 allowed).

**eTable3.** Assessment of selection bias (using a cohort study framework) in excluding patients with unknown sex or race and ethnicity.^1 2^

| **Selection probabilities**^3^ | | | |
| --- | --- | --- | --- |
|  | *Severe/Moderate disease* | *Mild ED/outpatient only* | *Selection Odds for exposed and unexposed* |
| *Phase 1* | α = *A° / A*  **0.03** | β = *B° / B*  **0.97** | r_E_ = α/β  0.03/0.97 = **0.03** |
| *Phase 2* | γ = *C° / C*  **0.07** | δ = *D° / D*  **0.93** | r_nE_ = $\gamma$/δ  0.07/0.93= **0.08** |

1. Based on methodology by Lash et al. (2009)[12]
2. Excluded patients n = 2,012
3. Note N3C data suppression rules (no cells < 20 allowed, observed data cells with < 20 denoted as “NA”), therefore raw cell counts are not shown here.

$$\frac{0.03 \left( \text{A}\text{ }\text{+}\text{ }\text{B} \right)\left( \text{ 0.08 × C}\text{ }\text{+}\text{ }\text{D} \right)}{0.08 \left( \text{C}\text{ }\text{+}\text{ }\text{D} \right)\left( \text{0.03 × A}\text{ }\text{+}\text{ }\text{B} \right)}\text{ }\text{- 1 = }\text{0 selection odds ratio}$$

**eTable4.** Full List of Variables and Concept Set Definitions Used to Form Final Patient Cohort.

| **VARIABLE** | **CONCEPTS AND LOGIC** |
| --- | --- |
| COVID-19 | **Concept Set Names (latest code set ID) with Description:**  ATLAS SARS-CoV-2 rt-PCR and AG (1000019497)  *Description: Includes 65 Concepts (Measurements), characterizing a positive PCR or Antigen test. Used in conjunction with* ResultPos (1000005668) *to ensure only positive results are used.*  N3C COVID DIAGNOSIS (1000012899)  *Description: Identify patients with a covid diagnosis. Includes Single ICD10CM code mapped to SNOMED CT.*  **Logic:**  Patient record must be associated with a positive SARS-CoV-2 lab test or N3C COVID Clinical Diagnosis. The above concept set is part of the N3C inclusion criteria for data submissions sent to N3C. |
| Influenza | **Concept Set Names (latest code set ID) with Description:**  ATLAS influenza PCR AG and diagnosis (334505959)  *Description: Includes 345 Concepts (267 Measurements and 78 Conditions), characterizing a positive PCR or Antigen test. Used in conjunction with ResultPos (1000005668) to ensure only positive results are used.*[28]  **Logic:**  Same logic as N3C COVID case identification, with measurements and conditions combined into a single concept set.  Link to full concept set elements on GitHub: <https://github.com/onaqvi11/covid-flu-peds-coinfection>. |
| Age groups | **Logic:**  We used age group based on two prior SARS-CoV-2 severity studies on children and adolescents:  [Deaths in Children and Adolescents Associated With COVID-19 and MIS-C in the United States](https://doi.org/10.1542%2Fpeds.2021-052273)  [SARS-CoV-2–Associated Deaths Among Persons Aged <21 Years — United States, February 12–July 31, 2020](http://dx.doi.org/10.15585/mmwr.mm6937e4)  The dataset containing age for patients is available in N3C here: [[LOGIC LIAISON TEMPLATE] L2 and L3 Fact Tables: All Patients](https://unite.nih.gov/workspace/module/view/latest/ri.workshop.main.module.3ab34203-d7f3-482e-adbd-f4113bfd1a2b?id=KO-BA3B835&view=focus)  Patients were categorized into four levels based on age at index encounter:   1) 0-4 years 2) 5-11 years 3) 12-17 years 4) 18-21 years |
| Race and ethnicity | **Logic:**  We used race and ethnicity categories based on guidance defined by the [National Institutes of Health.](https://grants.nih.gov/grants/guide/notice-files/not-od-15-089.html)  The dataset containing age for patients is available in N3C here: [[LOGIC LIAISON TEMPLATE] L2 and L3 Fact Tables: All Patients](https://unite.nih.gov/workspace/module/view/latest/ri.workshop.main.module.3ab34203-d7f3-482e-adbd-f4113bfd1a2b?id=KO-BA3B835&view=focus)  Patients were categorized into the following racial categories:   1) "Hispanic or Latino" for anyone with this race, otherwise 2) "Asian" 3) "Black or African American" 4) “Native Hawaiian or Other Pacific Islander” 5) "American Indian or Alaska Native" 6) "White" 7) or "Other" 8) "Unknown" is assigned to all others.   **NOTE:** A number of patients with a source value of Asian or Other Pacific Islander are categorized as “Unknown” until the site is able to differentiate and correct their source values since the current value does not reasonably fall into only one of the 6 minimum race/ethnicity categories defined by the NIH.  Patients were categorized into the following ethnicity categories:   1) "Hispanic or Latino Any Race" for anyone with this ethnicity 2) Otherwise "Asian Non-Hispanic" 3) "Black or African American Non-Hispanic" 4) “Native Hawaiian or Other Pacific Islander Non-Hispanic” 5) "American Indian or Alaska Native Non-Hispanic" 6) "White Non-Hispanic" 7) or "Other Non-Hispanic" 8) "Unknown" is assigned to all others   **NOTE:** A number of patients with a source value of Asian or Other Pacific Islander are categorized as “Unknown” until the site is able to differentiate and correct their source values since the current value does not reasonably fall into only one of the 6 minimum race/ethnicity categories defined by the NIH. |
| Pediatric Complex Chronic Conditions (PCCC) | **List of PCCC Categories:**   1) Cardiovascular 2) Congenital/genetic 3) Gastrointestinal 4) Hematologic/immunologic 5) Malignancy 6) Metabolic 7) Miscellaneous 8) Neonatal 9) Neurologic/neuromuscular 10) Renal 11) Respiratory (does not include asthma) 12) Technology dependent 13) Transplant   **Logic:**  We used a modified concept set of OMOP conditions and procedures using the Pediatric Complex Chronic Conditions (PCCC) translated from ICD10 CM and ICD10 PCS list developed by [Feudtner et al. (2014)](https://doi.org/10.1186/1471-2431-14-199).   1) Full list of 5,382 concepts available at: <https://github.com/onaqvi11/covid-flu-peds-coinfection>. |
| Asthma | **Concept Set Names (latest code set ID) with Description:**  [N3C] ASTHMA (456595451)  *Description: Includes 122 Concepts (121 Condition and 1 Observation) that broadly capture asthma as a condition including exacerbation*  **Logic:**  Identifies all patients with asthma or asthma exacerbation in the N3C. |
| Obesity | **Concept Set Names (latest code set ID) with Description:**  OBESITY (1000064350)  *Description: Includes 68 Concepts (60 Condition and 8 Observation) that broadly capture obesity diagnosis in N3C patients.*  **Logic:**  Broad and generic general use concept as starting place for research teams and for use in calculating comorbidity indices. **Used for patients aged 21 years only, for paediatric obesity definitions of patients 20 years and younger, see eMethods.** |
| Diabetes (types one or two) | **Concept Set Names (latest code set ID) with Description:**  DIABETES COMPLICATED (733797097) and DIABETES UNCOMPLICATED (1000052453)  *Description: Includes 565 Concepts (Conditions) that broadly capture diabetes diagnosis in N3C patients.*  **Logic:**  Create broad and generic general use concept as starting place for research teams and for use in calculating comorbidity indices. |
| Invasive mechanical ventilation | **Concept Set Names (latest code set ID) with Description:**  [ICU/MODS]IMV (1000047749)  **Logic:**  Includes patients associated with any procedure codes in the concept sets listed that had a procedure date during their COVID or influenza hospitalization. |
| Vasopressors/inotropic support | **Concept Set Names (latest code set ID) with number of concepts:**  [PASC] Vasopressors (791331225) = 76  **NOTE:** We updated logic to utilize a single concept set related to vasopressors/inotropic support that has been validated and used in other N3C studies as part of the RECOVER workspace.[29]  **Logic:**  Includes patients associated with any medication codes in the concept sets listed that had a medication administration date during their COVID or influenza hospitalization. |
| ECMO | **Concept Set Names (latest codes ID):**  Kostka – ECMO (415149730)  **Logic:**  Includes patients associated with any procedure codes in the concept sets listed that had a procedure date during their COVID of influenza hospitalization. |
| Death | **Logic:**  Patients were flagged as deceased if a valid entry with a date was included in the death table, which is part of the OMOP data model used in the Enclave. Patients were considered if they had a record of in-hospital death or discharge to hospice. |

**eTable5.** Characteristics of patients aged ≤ 21 years with COVID-19, influenza, and co-infection—National Clinical Cohort Collaborative (N3C), March 2020–April 2024.

| **Characteristic** | **No. of patients (%) with COVID-19 and influenza co-infection (n = 11,562)** | **No. of patients (%) with COVID-19-only (n = 1,208,609)** | **No. of patients (%) with influenza-only (n = 312,715)** |
| --- | --- | --- | --- |
| **Age group, yrs** | | | |
| 0-4 | 2,224 (19.2%) | 181,053 (15.0%) | 46,912 (15.0%) |
| 5-11 | 4,204 (36.4%) | 368,329 (30.5%) | 145,377 (46.5%) |
| 12-17 | 2,842 (24.6%) | 355,367 (29.4%) | 80,404 (25.7%) |
| 18-21 | 2,292 (19.8%) | 303,860 (25.1%) | 40,022 (12.8%) |
| **Sex** | | | |
| Male | 5,792 (50.1%) | 598,717 (49.5%) | 157,262 (50.3%) |
| Female | 5,419 (46.9%) | 584,495 (48.4%) | 148,060 (47.3%) |
| Unknown | 351 (3.0%) | 25,397 (2.1%) | 7,393 (2.4%) |
| **Race and ethnicity** | | | |
| American Indian or Alaska Native | 61 (0.5%) | 5,148 (0.4%) | 2,556 (0.8%) |
| Asian | 385 (3.3%) | 35,742 (3.0%) | 9,703 (3.1%) |
| Black/African American | 2,384 (20.6%) | 191,829 (15.9%) | 64,129 (20.5%) |
| Hispanic or Latino | 2,185 (18.9%) | 202,969 (16.8%) | 63,096 (20.2%) |
| Native Hawaiian or Other Pacific Islander | 46 (0.4%) | 3,318 (0.3%) | 1,292 (0.4%) |
| Other | 263 (2.3%) | 44,345 (3.7%) | 4,594 (1.5%) |
| White | 5,253 (45.4%) | 615,866 (51.0%) | 143,123 (45.8%) |
| Unknown | 985 (8.5%) | 109,392 (9.1%) | 24,222 (7.7%) |
| **Any pre-existing diagnosis** | | | |
| None | 7,159 (61.9%) | 819,959 (67.8%) | 171,690 (54.9%) |
| 1 condition | 3,010 (26.0%) | 263,595 (21.8%) | 92,460 (29.6%) |
| ≥ 2 conditions | 1,393 (12.0%) | 125,055 (10.3%) | 48,565 (15.5%) |
| **Specific pre-existing diagnoses^1^** | | | |
| Asthma | 1,164 (10.07%) | 97,780 (8.09%) | 44,589 (14.26%) |
| Cardiovascular | 377 (3.26%) | 36,283 (3.00%) | 12,465 (3.99%) |
| Congenital/Genetic | 587 (5.08%) | 52,354 (4.33%) | 19,166 (6.13%) |
| Diabetes (Type 1 or 2) | 92 (0.80%) | 9,263 (0.77%) | 2,284 (0.73%) |
| Gastrointestinal | 232 (2.01%) | 22,333 (1.85%) | 8,209 (2.63%) |
| Hematologic/Immunologic | 176 (1.52%) | 20,741 (1.72%) | 7,266 (2.32%) |
| Malignancy | 121 (1.05%) | 11,378 (0.94%) | 3,274 (1.05%) |
| Metabolic | 354 (3.06%) | 33,050 (2.73%) | 10,929 (3.49%) |
| Neonatal | 161 (1.39%) | 15,541 (1.29%) | 5,519 (1.76%) |
| Neurologic/Neuromuscular | 488 (4.22%) | 47,500 (3.93%) | 15,582 (4.98%) |
| Obesity | 2,574 (22.26%) | 228,370 (18.90%) | 80,958 (25.89%) |
| Renal | 224 (1.94%) | 20,616 (1.71%) | 8,045 (2.57%) |
| Respiratory | 290 (2.51%) | 25,274 (2.09%) | 10,422 (3.33%) |
| Technology Dependent | 148 (1.28%) | 15,957 (1.32%) | 4,821 (1.54%) |
| **Disease severity** | | | |
| Mild (outpatient visit only) | 6,485 (56.1%) | 923,854 (76.4%) | 194,225 (62.1%) |
| Mild ED (outpatient with emergency department visit) | 4,372 (37.8%) | 236,057 (19.5%) | 106,615 (34.1%) |
| Moderate (hospitalised without requiring vasopressors/inotropes, invasive ventilation, or ECMO) | 618 (5.3%) | 43,471 (3.6%) | 10,999 (3.5%) |
| Severe (hospitalised and requiring vasopressors/inotropes, invasive ventilation, or ECMO, or death) | 87 (0.8%) | 5,227 (0.4%) | 876 (0.3%) |

1. Note that patients with a history of transplant were omitted due to N3C data suppression rules (no cells < 20 allowed) due to the potential for back calculation.

**eTable6.** Frequency and proportion of patients aged ≤ 21 years with COVID-19 and influenza co-infection, by influenza season and SARS-CoV-2 variant period of predominance—National Clinical Cohort Collaborative (N3C), March 2020–April 2024.

| **No. (%) of co-infected patients** | **Influenza Season** | | | | |
| --- | --- | --- | --- | --- | --- |
|  | *2019-2020* | *2020-2021* | *2021-2022* | *2022-2023* | *2023-2024* |
|  | 26 (0.25%) | 534 (5.2%) | 4,187 (40.74%) | 3,049 (29.67%) | 2,481 (24.14%) |
|  | **SARS-CoV-2 variant period of predominance** | | | | |
|  | *Wild-Type* | *Delta* | *Omicron-BA* | *Omicron-XBB* | *Omicron-JN.1* |
|  | 558 (5.43%) | 762 (7.41%) | 6,119 (59.54%) | 1,773 (17.25%) | 1,065 (10.36%) |

**eTable7.** Frequency and proportion of clinical outcomes among patients aged ≤ 21 years with COVID-19 and influenza, by COVID-19 pandemic phase^1^—National Clinical Cohort Collaborative (N3C), March 2020–April 2024.

| **COVID-19-only** | | | |
| --- | --- | --- | --- |
| **Disease severity** | **No. of patients (%) during Phase 1 (n = 683,644)** | **No. of patients (%) during Phase 2 (n = 295,196)** | **Crude^2 3^ p-value** |
| Mild (outpatient visit only) | 641,142 (81.8%) | 178,082 (60.3%) | < 0.001 |
| Mild ED (outpatient with emergency department visit) | 117,494 (15.0%) | 98,020 (33.2%) |  |
| Moderate (hospitalised without requiring invasive ventilation, or ECMO) | 22,446 (2.9%) | 16,937 (5.7%) |  |
| Severe (hospitalised and requiring invasive ventilation, or ECMO, or death) | 2,562 (0.3%) | 2,157 (0.7%) |  |
| **Influenza-only** | | | |
| **Disease severity** | **No. of patients (%) during Phase 1 (n = 44,422)** | **No. of patients (%) during Phase 2 (n = 237,697)** | **Crude^2 3^ p-value** |
| Mild (outpatient visit only) | 30,383 (68.4%) | 142,688 (60.0%) | < 0.001 |
| Mild ED (outpatient with emergency department visit) | 12,625 (28.4%) | 85,392 (35.9%) |  |
| Moderate (hospitalised without requiring invasive ventilation, or ECMO) | 1,275 (2.9%) | 8,964 (3.8%) |  |
| Severe (hospitalised and requiring invasive ventilation, or ECMO, or death) | 139 (0.3%) | 653 (0.3%) |  |

1. Phase 1 was defined as 1 March 2020 to 31 March 2022; Phase 2 included the period from 1 April 2022 to 30 April 2024.
2. Univariate differences were compared using GEE models with a binomial family for categorical variables and a log link function. All models accounted for clustering by data partners. All global p-values represent comparisons between the variables during the “acute” or “post-acute” pandemic phase, among co-infected patients.
3. Patients with unknown sex or race and ethnicity excluded from statistical testing (n = 129,769 for COVID-19-only and n = 30,596 for influenza-only).

**eTable8.** Sensitivity, specificity, and positive predictive value of varying COVID-19 and influenza co-infection case definitions among patients aged ≤ 21 years—National Clinical Cohort Collaborative (N3C), March 2020–April 2024.**^1^**

| **Co-infection case definition level (laboratory results and diagnostic codes)^2^** | **Sensitivity** | **Specificity** | **Positive Predictive Value** |
| --- | --- | --- | --- |
| Same Day (n = 9,062) | 1.00 | 0.999 | 0.876 |
| 3 days apart (n = 10,067) | 1.00 | 0.998 | 0.788 |
| 7 days apart (n = 10,277)^3^ | 1.00 | 0.998 | 0.772 |
| 10 days apart (n = 10,420) | 1.00 | 0.998 | 0.761 |
| 14 days apart (n = 10,691) | 1.00 | 0.997 | 0.742 |

1. Data partner, sex, and race and ethnicity exclusions applied prior to calculations.
2. All calculations compared to COVID-19 and influenza positive laboratory result only, documented zero days apart (Gold Standard; n = 7,934).
3. Default chosen study case definition.

**eFigure1.** Sensitivity analysis of modelled Severe/Moderate disease^1^ versus mild ED/outpatient disease^2^ associated with COVID-19 and influenza co-infection, patients aged ≤ 21 years, by age group and number of pre-existing diagnoses, by co-infection case definition—National Clinical Cohort Collaborative (N3C), March 2020–April 2024.^3 4^

1. Severe/Moderate disease is defined as: hospitalised without critical care support, or hospitalised and requiring invasive ventilation, vasopressors/inotropes, or ECMO, or death.
2. Mild ED/outpatient is defined as: outpatient visit only or outpatient with emergency department visit.
3. Risk ratios were compared using GEE models with a binomial family for categorical variables and a log link function. All models accounted for clustering by data partners and used a change-in-estimate approach implemented through backward elimination; the final model included age group and number of pre-existing diagnoses.
4. Age Group 18-21 and No conditions are reference.

**E-References**

1.	Benchimol EI, et al. The REporting of studies Conducted using Observational Routinely-collected health Data (RECORD) statement. PLoS Medicine. 2015;12(10):e1001885.

2.	Pfaff E. COVID-19 Phenotype Documentation, Version 4.0: N3C Phenotype & Data Acquisition Workstream; 2022 [Available from: https://github.com/National-COVID-Cohort-Collaborative/Phenotype_Data_Acquisition/wiki/Latest-Phenotype.]

3.	Hardin AP, Hackell JM, PRACTICE CO, MEDICINE A, Simon GR, Boudreau ADA, et al. Age Limit of Pediatrics. Pediatrics. 2017;140(3).

4.	Haendel MA, et al. The National COVID Cohort Collaborative (N3C): Rationale, design, infrastructure, and deployment. Journal of the American Medical Informatics Association. 2020;28(3):427-43.

5.	Bennett TD, et al. Clinical Characterization and Prediction of Clinical Severity of SARS-CoV-2 Infection Among US Adults Using Data From the US National COVID Cohort Collaborative. JAMA Network Open. 2021;4(7):e2116901.

6.	National Center for Advancing Translational Sciences. N3C Frequently Asked Questions: NCATS N3C Data Enclave; [Available from: https://covid.cd2h.org/faq/.]

7.	Hayden MK, et al. Infectious Diseases Society of America Guidelines on the Diagnosis of Coronavirus Disease 2019: Serologic Testing. Clinical Infectious Diseases. 2024.

8.	Leese P, et al. Clinical encounter heterogeneity and methods for resolving in networked EHR data: a study from N3C and RECOVER programs. Journal of the American Medical Informatics Association. 2023;30(6):1125-36.

9.	Antoon JW, et al. Accuracy of Influenza ICD-10 Diagnosis Codes in Identifying Influenza Illness in Children. JAMA Network Open. 2024;7(4):e248255-e.

10.	Agathis NT, et al. Codetections of Other Respiratory Viruses Among Children Hospitalized With COVID-19. Pediatrics. 2023;151(2).

11.	Stowe J, et al. Interactions between SARS-CoV-2 and influenza, and the impact of coinfection on disease severity: a test-negative design. International Journal of Epidemiology. 2021;50(4):1124-33.

12.	Lash TL, Fink AK, Fox MP. Selection Bias. In: Lash TL, Fox MP, Fink AK, editors. Applying Quantitative Bias Analysis to Epidemiologic Data. New York, NY: Springer New York; 2009. p. 43-57.

13.	Kamidani S, et al. Epidemiology, Clinical Characteristics, and Outcomes of Influenza-Associated Hospitalizations in US Children Over 9 Seasons Following the 2009 H1N1 Pandemic. Clinical Infectious Diseases. 2022;75(11):1930-9.

14.	Feudtner C, et al. Pediatric complex chronic conditions classification system version 2: updated for ICD-10 and complex medical technology dependence and transplantation. BMC Pediatrics. 2014;14:199.

15.	Applicable Data Methods & Standards Domain Team. N3C Concept Set - 456595451 ([N3C] ASTHMA): Zenodo; 2024 [Available from: https://doi.org/10.5281/zenodo.11194018.]

16.	Hampl SE, et al. Clinical Practice Guideline for the Evaluation and Treatment of Children and Adolescents With Obesity. Pediatrics. 2023;151(2).

17.	Applicable Data Methods & Standards Domain Team. N3C Concept Set - 581513221 (OBESITY): Zenodo; 2024 [Available from: https://doi.org/10.5281/zenodo.11194167.]

18.	O’Neil ST, et al. The Researcher’s Guide to N3C: A National Resource for Analyzing Real-World Health Data. 2023.

19.	Applicable Data Methods & Standards Domain Team. N3C Concept Set - 248468138 (DIABETES UNCOMPLICATED): Zenodo; 2024 [Available from: https://doi.org/10.5281/zenodo.11194108.]

20.	Centers for Disease Control and Prevention (CDC). CDC Growth Charts: National Center for Health Statistics; [Available from: https://www.cdc.gov/growthcharts/extended-bmi.htm.]

21.	Centers for Disease Control and Prevention (CDC). Child and Teen BMI Categories: National Center for Chronic Disease Prevention and Health Promotion (NCCDPHP); Division of Nutrition, Physical Activity, and Obesity; [Available from: https://www.cdc.gov/bmi/child-teen-calculator/bmi-categories.html.]

22.	Centers for Disease Control and Prevention (CDC). Influenza Activity in the United States during the 2022–2023 Season and Composition of the 2023–2024 Influenza Vaccine: National Center for Immunization and Respiratory Diseases (NCIRD); 2023 [Available from: https://www.cdc.gov/flu/whats-new/22-23-summary-technical-report.html.]

23.	Centers for Disease Control and Prevention (CDC). COVID Data Tracker Atlanta, GA: U.S. Department of Health and Human Services, CDC; 2024 [Available from: https://covid.cdc.gov/covid-data-tracker.]

24.	Centers for Disease Control and Prevention (CDC). COVID-NET: National Center for Immunization and Respiratory Diseases; Coronavirus and Other Respiratory Viruses Division; 2024 [Available from: https://www.cdc.gov/covid/php/covid-net/index.html#:~:text=A%20case%20is%20defined%20as,days%20before%20or%20during%20hospitalization.]

25.	Centers for Disease Control and Prevention (CDC). Influenza Hospitalization Surveillance Network (FluSurv-NET): National Center for Immunization and Respiratory Diseases (NCIRD); 2024 [Available from: https://www.cdc.gov/flu/weekly/influenza-hospitalization-surveillance.htm.]

26.	Martin B, et al. Characteristics, Outcomes, and Severity Risk Factors Associated With SARS-CoV-2 Infection Among Children in the US National COVID Cohort Collaborative. JAMA Network Open. 2022;5(2):e2143151-e.

27.	Højsgaard S, Halekoh U, Yan J. The R Package geepack for Generalized Estimating Equations. Journal of Statistical Software. 2005;15(2):1 - 11.

28.	Naqvi OH, Beasley WH, Tyungu DL, Cheng JJ, Wendelboe AM. N3C Concept Set - 334505959 (Influenza measurement and conditions) (Version 6): Zenodo; 2025 [Available from: https://doi.org/10.5281/zenodo.15185586.]

29.	Hill EL, et al. Risk factors associated with post-acute sequelae of SARS-CoV-2: an N3C and NIH RECOVER study. BMC Public Health. 2023;23(1):2103.
